# Efficient Transfer of Chirality in Complex Hybrid Materials and Impact on Chirality-induced Spin Selectivity

**DOI:** 10.1021/acs.chemmater.4c02108

**Published:** 2024-11-20

**Authors:** Md Anik Hossain, Sara Illescas-Lopez, Md Wazedur Rahman, Mari C. Mañas Torres, Rafael Contreras-Montoya, Seyedamin Firouzeh, José A. Gavira, Luis Álvarez de Cienfuegos, Sandipan Pramanik

**Affiliations:** † Department of Electrical and Computer Engineering, University of Alberta, Edmonton, AB T6G 1H9, Canada; ‡ Departamento de Química Orgánica, Unidad de Excelencia Química Aplicada a Biomedicina y Medioambiente (UEQ), Universidad de Granada (UGR), C. U. Fuentenueva, Avda. Severo Ochoa s/n, E-18071 Granada, Spain; § National Research Council Canada, Edmonton, AB T6N 1E4, Canada; ∥ Nanoscopy-UGR Laboratory, Facultad de Farmacia, Campus de Cartuja, 18071 Granada, Spain; ⊥ Laboratorio de Estudios Cristalográficos, Instituto Andaluz de Ciencias de la Tierra (Consejo Superior de Investigaciones Científicas), Avenida de las Palmeras 4, Armilla, 18100 Granada, Spain; # Instituto de Investigación Biosanitaria ibs.GRANADA, Av. de Madrid, 15, 18016 Granada, Spain

## Abstract

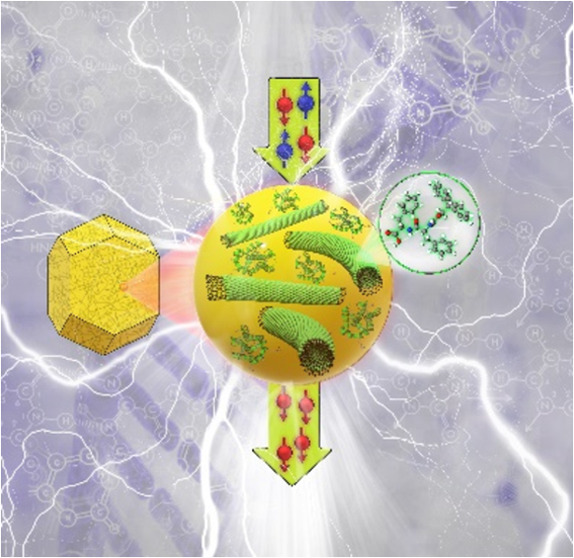

Transfer of chirality, or transmission of asymmetric information from one system to another, plays an essential role in fundamental biological and chemical processes and, therefore, is essential for life. This phenomenon also holds immense potential in spintronics in the context of chirality-induced spin selectivity (CISS). In the CISS, the spatial arrangement of chiral molecules influences the spin state of electrons during the charge-transfer processes. Transfer of chirality from chiral molecules to an achiral material in a hybrid environment enables induction of spin polarization in the achiral material, thus vastly expanding the library of CISS-active electronic materials. Such “induced” CISS signals could have different responses compared to pure chiral molecules because the electronic properties of the achiral material come into play in the former case. In addition, multiple chiral sources can be used, which can have a nontrivial contribution to the induced CISS effect and can act either synergistically or antagonistically. This opens the way to achieving tunability of the CISS signals via chemical means. Earlier, such a chirality-transfer phenomenon and the resulting induced CISS effect were demonstrated in a hybrid system containing carbon nanotubes (CNTs) functionalized with a chiral agent (Fmoc-diphenylalanine l/d). In this context, we extend this result by investigating the role of an additional chiral moiety (l-lysozyme enzyme crystals) in this system. Here, the chiral crystal surrounds the chiral-functionalized CNTs, and we show that synergistic interactions result in more efficient chirality transfer, resulting in nontrivial changes in the CISS effect. This manifests in the form of (a) a stronger CISS signal compared to only one single chiral agent, (b) nonmonotonic temperature dependence and sign reversal of the CISS signal, and (c) persistence of the CISS signal at higher temperatures. Hybrid chiral materials with multiple chiral sources could, therefore, offer intricate control of the CISS signal via modification of its constituents, which is not possible in homogeneous chiral systems with single chiral sources.

## 1 Introduction

Short-peptide supramolecular hydrogels are advanced materials that have found use in a multitude of biological and technological applications.^1−3^ These materials are formed by the noncovalent self-assembly of aromatic short peptides, giving rise to fibers that intertwine to form a network that can immobilize water at a macroscopic level, giving rise to supramolecular gels. The hydrophobic and amphiphilic characteristics of these peptides have made them useful as a solvate and to disperse a multitude of metallic, inorganic, and carbon-based materials, giving rise to composite or hybrid materials.^4−8^ Furthermore, their mild forming condition along with their suitable mechanical and diffusion properties have made these materials useful in cell culture and tissue engineering and as a medium for growing organic and inorganic crystals.^8−13^ Being chiral, the self-assembly of these peptides gives rise to fibers that present supramolecular chirality. Particularly relevant is to study how this supramolecular chirality can modulate the resulting properties of the hydrogels and to what extent can it be transferred to other organic and inorganic materials to obtain composite materials with new or improved chiral properties.^14^ This strategy has been used to obtain an increase in the chiroptical response by transferring chirality to solid nanoparticles.^15^ For example, the transfer of chirality to gold nanoparticles has been carried out by growing gold nanoparticles in a helical supramolecular polymer^16^ and by trapping them in the process of self-assembly.^17^ Recently, perovskite nanocrystals trapped in a chiral supramolecular network have shown circularly polarized luminescence, indicating that this approach can be useful in the design of novel chiroptical materials.^18^

Another field that has benefited from this transfer of chirality is organic spintronics through the consecution of chiral hybrid materials showing chirality-induced spin selectivity (CISS).^19^ This effect shows that there is a correlation between the chirality of the medium and the carrier spin and, therefore, could be key in the area of nanospintronics.^20,21^ Moreover, the possibility to analyze the behavior of chiral molecules or materials through this phenomenon has brought a new degree of freedom in the chemistry world, showing that the CISS effect can have applications in chiral separation, recognition, detection, and asymmetric catalysis.^22^ CISS has been reported in a wide variety of chiral molecules^20^ as well as in some chiral hybrid materials, such as hybrid organic–inorganic perovskites, chiral molecule intercalated van der Waals materials, etc.^19^ Recently, we have shown that biomolecules such as DNA and short-peptide supramolecular hydrogels based on Fmoc-FF (Fmoc-diphenylalanine) are able to transfer their supramolecular chirality to carbon nanotubes (CNTs) and nanographenes, thus endowing them with CISS properties.^23−31^ In these examples, CNTs are surrounded by chiral DNA and peptide fibers, respectively, that are in close contact with the CNT surface. Nevertheless, herein, we wondered if this process of transfer of supramolecular chirality could be further modulated or improved by the incorporation of another chiral source that could also interact supramolecularly with the CNT, avoiding disruption of the electronic behavior of the nanotube. In such a system, we could study how effective chirality transfer would be in terms of proximity and type of molecule and whether this effect could be additive or counterproductive based on the CISS values.

**Figure 1:**
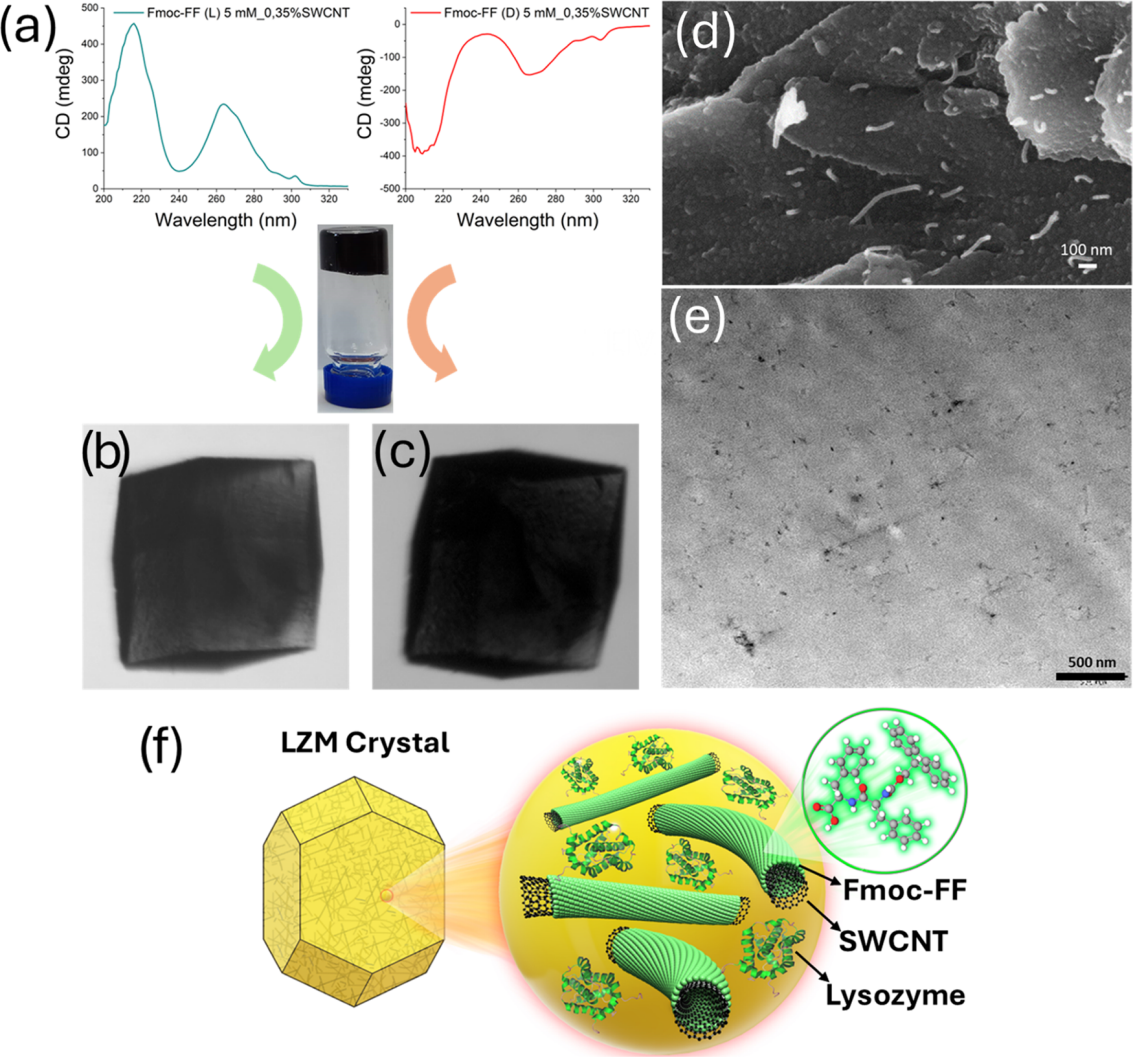
(a) Circular dichroism (CD) spectra of Fmoc-FF + SWCNTs solutions (green, L chirality; red, D chirality). (b) SWCNT@CLLC grown in Fmoc-FF (L) and (c) SWCNT@CLLC grown in Fmoc-FF (D). (d) Scanning electron microscopy (SEM) image of the interior of SWCNT@CLLC, where white filaments are bundles of CNTs. (e) Transmission electron microscopy (TEM) image of the interior of SWCNT@CLLC, black spots are bundles of CNTs. (f) Schematic representation of the composite chiral hybrid materials in which CNTs are surrounded by two chiral compounds arranged supramolecularly.

To explore these possibilities, we used single walled carbon nanotube (SWCNT)-loaded cross-linked LZM crystals (SWCNT@CLLC). Fabrication of SWCNT@CLLC was reported previously via the in situ growth of lysozyme (LZM) crystals in hybrid hydrogels of Fmoc-FF-coated CNTs.^32^ We showed that these composite crystals were able to conduct electricity through the CNTs contained in their interior.^32^ Considering that proteins in their crystalline form also have a supramolecular arrangement, these materials are ideal for studying the CISS effect in complex three-dimensional (3D) ordered systems, in which CNTs experience two supramolecular chiral environments (peptide fibers and proteins) simultaneously (Figure 1f). To study the influence of both chiral sources, we have obtained LZM crystals grown in Fmoc-FF (L and D) hydrogels as well as in Fmoc-GG (diglycine) hydrogels in which the only chiral source is the protein (Figure 1). Results reported in this work shed light on the chirality-transfer process in hybrid materials for spintronics applications and can help understand in more detail the relationship between these two effects and contribute to the development of novel materials with superior spintronics applications.

## 2 Results and Discussion

SWCNT@CLLCs were fabricated using the protocol described in ref (32) (see Experimental Section). Since these crystals are grown inside an already prepared Fmoc-dipeptide + SWCNT gel, the SWCNTs have two layers attached to their walls: the inner layer is the peptide and the outer layer is LZM, as shown schematically in Figure 1f. Thus, the CNTs are subjected to two chiral environments: (a) l- or d-chiral Fmoc-FF and (b) and l-chiral LZM. We have tested SWCNT@CLLC samples with varying SWCNT concentrations of 0.1, 0.25, 0.5, and 0.7 mg/mL. The role of peptide chirality has been tested by replacing Fmoc-FF with achiral Fmoc-GG (diglycine). We also tested two types of control samples: LZM crystals grown in water (no Fmoc-FF or SWCNT) and LZM crystals grown in Fmoc-FF without SWCNT. In this work, CNTs are ∼95% semiconducting with ∼41% having (6,5) chirality; ≥95% carbon basis, i.e., ≥95% as carbon nanotubes; 0.78 nm average diameter; CAS: 308068-56-6, provided by Sigma- Aldrich: https://www.sigmaaldrich.com/ES/es/product/aldrich/773735. In our earlier work on Fmoc-FF-functionalized CNTs, we have shown that the CISS signal correlates with the supramolecular chirality rather than atomic or molecular scale chirality, so CNT chirality should not affect the CISS signal.^28^

**Figure 2:**
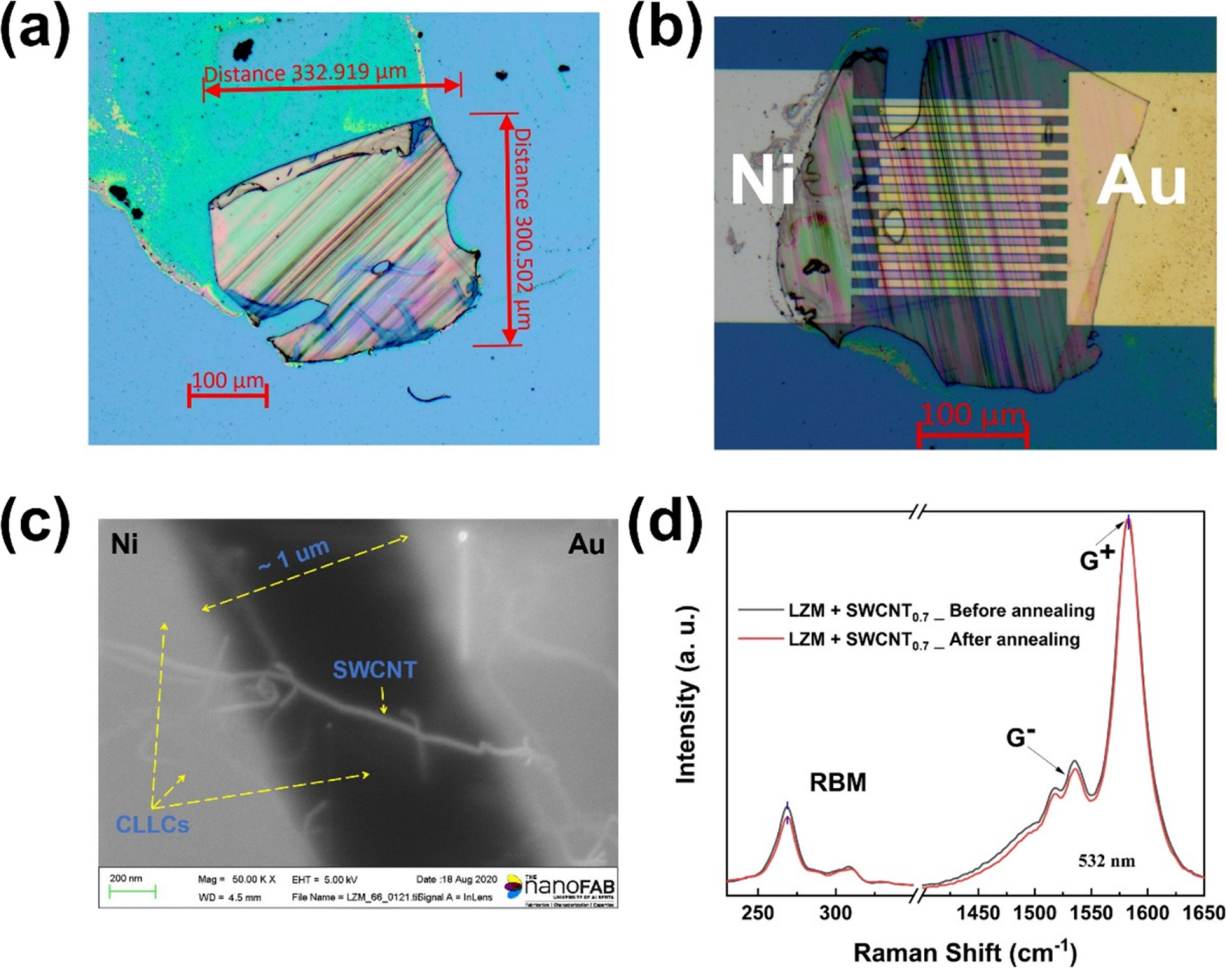
(a, b) Optical microscopy images of a typical slice of SWCNT@CLLC placed on a SiO_2_ substrate and interdigitated Ni/Au electrodes, respectively. (c) Field emission scanning electron microscope (FESEM) image of a SWCNT@CLLC (SWCNT concentration: 0.7 mg/mL, tubes coated with Fmoc-FF-L) slice placed on top of prepatterned Ni–Au electrodes. (d) Room-temperature Raman characterization of SWCNT@CLLC (SWCNT concentration: 0.7 mg/mL, Fmoc-FF-L coated). Signature Raman peaks are indicated with no significant change because of annealing.

Figure 2a shows a typical slice (∼900 nm) of SWCNT@CLLC on a SiO_2_ substrate. For electrical measurements, such slices are transferred between a pair of interdigitated electrodes, as shown in Figure 2b. A SEM image of the device is shown in Figure 2c, in which the white strands indicate interconnected SWCNTs, embedded within the composite protein crystal and extending between the two electrodes. The devices are vacuum annealed to improve electrical contacts. Figure 2d shows a representative Raman spectrum (532 nm excitation, room temperature) before and after annealing of the sections. Two main features of SWCNTs can be seen: (a) radial breathing mode (RBM) occurring at lower wavenumbers (240–280 cm^–1^) and (b) tangential mode (TM) or G^+^ and G^–^ mode occurring at higher wavenumbers (1500–1600 cm^–1^).^33^ Annealing does not appear to have any adverse effect on the nanotube crystal structure or its interaction with the surrounding chiral medium. The Raman features of SWCNT@CLLC samples as well as SWCNT + Fmoc-FF samples (i.e., without any LZM) have been studied in detail in our previous works.^27,28,32^ Fmoc-FF causes an upshift in the location of the G^+^ band relative to bare CNTs, and this upshift is further enhanced for SWCNT@CLLCs, i.e., when LZM is present.^32^ The same trend is observed for the G^–^/G^+^ intensity ratio, which decreases systematically from bare CNTs to Fmoc-FF + CNTs^27^ to SWCNT@CLLC. In Figure 2d, the G^–^/G^+^ ratio is ≈32%, whereas that for Fmoc-FF + CNTs is ≈39%. The G^–^ peak originates from the circumferential vibration of the carbon atoms because of the cylindrical geometry of CNTs.^33^ Molecular attachments on the CNT walls tend to suppress this vibration and reduce the intensity of the G^–^ peak. Thus, all of these features indicate a synergistic interaction of both chiral molecules with the SWCNTs. This synergy can occur in the form of direct interaction of each type of molecule with the SWCNT, or enhanced interaction of the Fmoc-FF with SWCNT due to a compression exerted by the outer protein layer in its crystalline form. Given the nature of the present samples, as illustrated schematically in Figure 1f, the latter mechanism is more likely.

**Figure 3:**
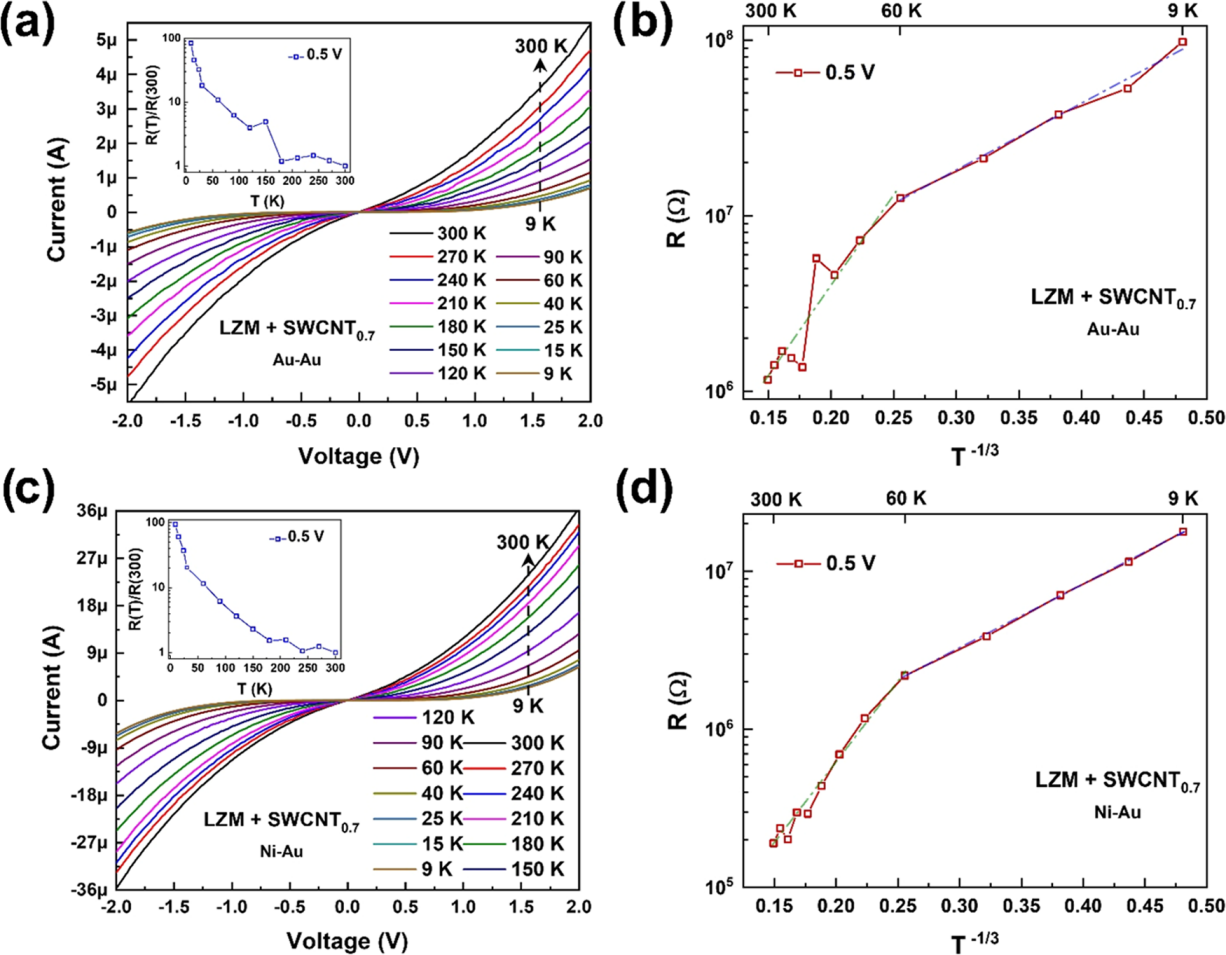
Current–voltage (*I*–*V*) characteristics at *B* = 0 and fitting with the VRH model with *d* = 2 for SWCNT@CLLC (SWCNT concentration: 0.7 mg/mL, tubes coated with Fmoc-FF-L) samples. (a, b) Au–Au electrodes and (c, d) Ni–Au electrodes. The insets in parts (a) and (c) show normalized resistance, measured at 0.5 V, as a function of temperature.

While the electrical transport properties of Fmoc-FF+SWCNTs have been reported before,^27,28^ those of the SWCNT@CLLC crystals have not. Also, considering the differences in the Raman responses discussed above, it is necessary to explore whether these differences translate to the electrical properties of SWCNT@CLLC or not. For this purpose, we perform temperature (*T*)-dependent (9–300 K) current–voltage (*I*–*V*) measurements on these samples using Au–Au and Ni–Au contacts under a zero magnetic field. Figure 3 shows data from 0.7 mg/mL SWCNT samples, which exhibit semiconducting temperature dependence. Data from 0.5 mg/mL SWCNT samples are presented in the Supporting Information (Figure S1), which also exhibits the same temperature dependence. The contact resistances are ∼6 orders of magnitude smaller than the actual devices, and unlike the actual samples, they show metallic temperature dependence, and hence their role in the electrical measurements is negligible. Control samples with no SWCNT content (both LZM and LZM + Fmoc-FF) and low SWCNT content (0.1 and 0.25 mg/mL) show currents that are below the detection level of our measurement setup. Therefore, in this work, we report data from samples with SWCNT concentrations of 0.5 and 0.7 mg/mL. This indicates that the chiral matrix does not directly participate in electron conduction, at least not on the length scale (∼2 μm) used in this experiment. The absence of significant conductance from lower nanotube concentration samples indicates the absence of interconnecting nanotube networks extending between the electrodes in such samples. Sections with different thicknesses (100, 300, 500, 700, and 900 nm) were also tested using 0.5 and 0.7 mg/mL SWCNT samples. Measurable current values were obtained only from 700 and 900 nm thick samples, indicating that for the thinner slices, nanotube networks are below the conduction percolation threshold. In this paper, we will report data from 900 nm thick samples.

Figure 3a,c shows the *I*–*V* data from samples with 0.7 mg/mL SWCNT using Au–Au and Ni–Au contacts, respectively. Transport is nonlinear over the measured bias range. The insets show orders of magnitude decrease in device resistance *R* as the sample temperature is increased (i.e., d*R*/d*T* < 0). Such strong temperature dependence is indicative of phonon-assisted hopping and is a signature feature of SWCNT networks, whether chiral-functionalized^26−28^ or not.^34−36^ Nanotube networks can be viewed as conductive regions separated (or “localized”) by electrical “barriers” arising from tangled regions, intertube contacts, or tube defects.^37^ Carrier transmission between these localized states is mediated by phonon absorption or emission.^38^

This leads to diffusion-type finite conductivity, which is typically described using the variable-range hopping (VRH) model^39^*R*(*T*) = *R*_0_ exp(*T*_0_/*T*)^1/*d*+1^, where the prefactor *R*_0_ is determined by electron–phonon interaction, *d* depends on the dimensionality of the system, and *T*_0_ is a parameter inversely dependent on localization length ξ (decay length of a localized carrier’s wave function). For two-dimensional systems without electron–electron interaction, the exponent 1/(*d* + 1) is typically 1/3 (i.e., *d* = 2) and *T*_0_ ≈ 13/*g*ξ^2^, where *g* is the density of the states at the Fermi level.^39,40^ If electron–electron interaction is present, the exponent is 1/2 (i.e., *d* = 1) and *T*_0_ ≈ *e*^2^/κξ where *e* is the electronic charge and κ is the dielectric constant.^35,39^Figure 3b,d shows the fittings with the VRH model for *d* = 2. Additional fitting with *d* = 1 has been shown in Figure S2. A linear fit is observed over a wide temperature range (60–300 K). At lower temperatures (9–60 K), a linear fit is still observed, albeit with a smaller slope, consistent with the chiral-functionalized SWCNT samples studied before.^26−28^ As seen from Figure S1, SWCNT@CLLC (0.5 mg/mL) samples also qualitatively show the same features. Summarizing the above, despite the differences observed in Raman behavior due to the presence of the protein crystals, such differences did not translate to the electrical properties of the SWCNTs. This is consistent with the schematic shown in Figure 1f, according to which electrical transport through SWCNTs should be dictated by the molecular layer attached directly to the CNT walls, i.e., Fmoc-FF. Whether the presence of LZM on the outer layer leads to any difference in the CISS properties will be explored next.

Next, we proceeded with magnetic field (***B***)-dependent measurements. It is to be noted that in all magnetoresistance (MR) studies described below, we adopted a longitudinal configuration, in which ***B*** || ***I***. This is consistent with standard practices used for pure chiral systems in a vertical sandwich geometry.^20^ It is important to note that SWCNT-based planar hybrid systems (such as SWCNTs functionalized with Fmoc-FF) have been studied by us before.^27,28,30^ The CISS signal has been found to depend on the measurement angle between **B** and ***I***, and there is always a difference between the magnitudes of CISS responses of two chiralities. It was found that the opposite sign of the CISS signal for opposite chiralities appears in a transverse configuration, where ***B*** is perpendicular to ***I***. In the longitudinal configuration, however, both l- and d-chiralities have the same sign, albeit with different magnitudes. The magnitude of the CISS signal has been found to be stronger in the longitudinal geometry. As discussed below, the samples described above typically show low signal-to-noise ratios, which prompted us to choose the longitudinal configuration, where the CISS signal is stronger,^27,28^ even though it does not show opposite signs for opposite chiralities.

**Figure 4:**
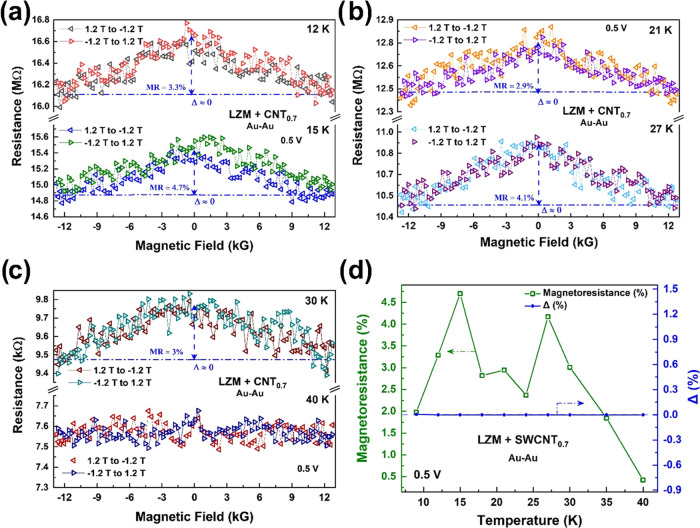
(a–c) Symmetric MR of SWCNT@CLLC crystals (SWCNT concentration: 0.7 mg/mL, tubes coated with Fmoc-FF-L) with Au–Au contacts at representative temperatures (applied bias is 0.5 V in all cases). Note the nonmonotonicity of the MR values as the temperature increases. (d) Temperature dependence of the background MR. MR asymmetry, Δ = 0 at all temperatures.

Figure 4a–c shows the representative MR^a^ data from SWCNT@CLLC (0.7 mg/mL) samples in the range of ±12 kG, measured at 0.5 V bias, using nonmagnetic Au–Au contacts. A negative background MR (∼4%) is observed, which is symmetric with respect to the magnetic field direction, i.e., *R*(+12 kG) – *R*(−12 kG) = Δ = 0, at all temperatures (Figure 4d). Particularly noteworthy from Figure 4a–c is the nonmonotonic temperature evolution of the background MR. As summarized in Figure 4d, a “double peak” feature is observed in the background MR response: the first peak occurs in the 10–20 K range and the next in the 20–40 K range. The background MR disappears at higher temperatures (>40 K) in the measured field range. Multiple samples have been tested, and they all exhibit the same behavior. Since Au contacts are nonmagnetic, they are unable to detect the presence of any chirality-induced spin polarization of the charge carriers in the nanotubes; hence, the overall MR is symmetric. We note that such negative MR is a signature feature of bare SWCNT networks and has been observed before by other groups;^34,35,41−43^ however, no nonmonotonic temperature dependence was reported. Such a nonmonotonicity is only observed when the SWCNTs are functionalized by chiral molecules.^26,27^

To detect the presence of any CISS-induced spin polarization due to the chiral media surrounding the nanotubes, we have replaced one of the Au contacts with a ferromagnetic Ni contact, which serves as a spin detector. Such a two-terminal detection of spin polarization is generally precluded in the linear-response regime,^44^ with possible exceptions when chiral molecules are present.^45^ No such restriction exists in nonlinear regime^44,45^ and has been commonly employed to detect CISS-induced spin polarization in molecular systems.^20^ Several mechanisms have been proposed to explain such a two-terminal detection. For example, ref (46) noted that energy-dependent transport and energy relaxation (e.g., phonon-activated conduction) are required for such detection to take place. According to ref,^45^ in a two-terminal configuration with only one magnetic electrode, true equilibrium is reached with local CISS-induced spin accumulation in the nonmagnetic electrode. Magnetization reversal leads to an asymmetric MR in such cases.

**Figure 5:**
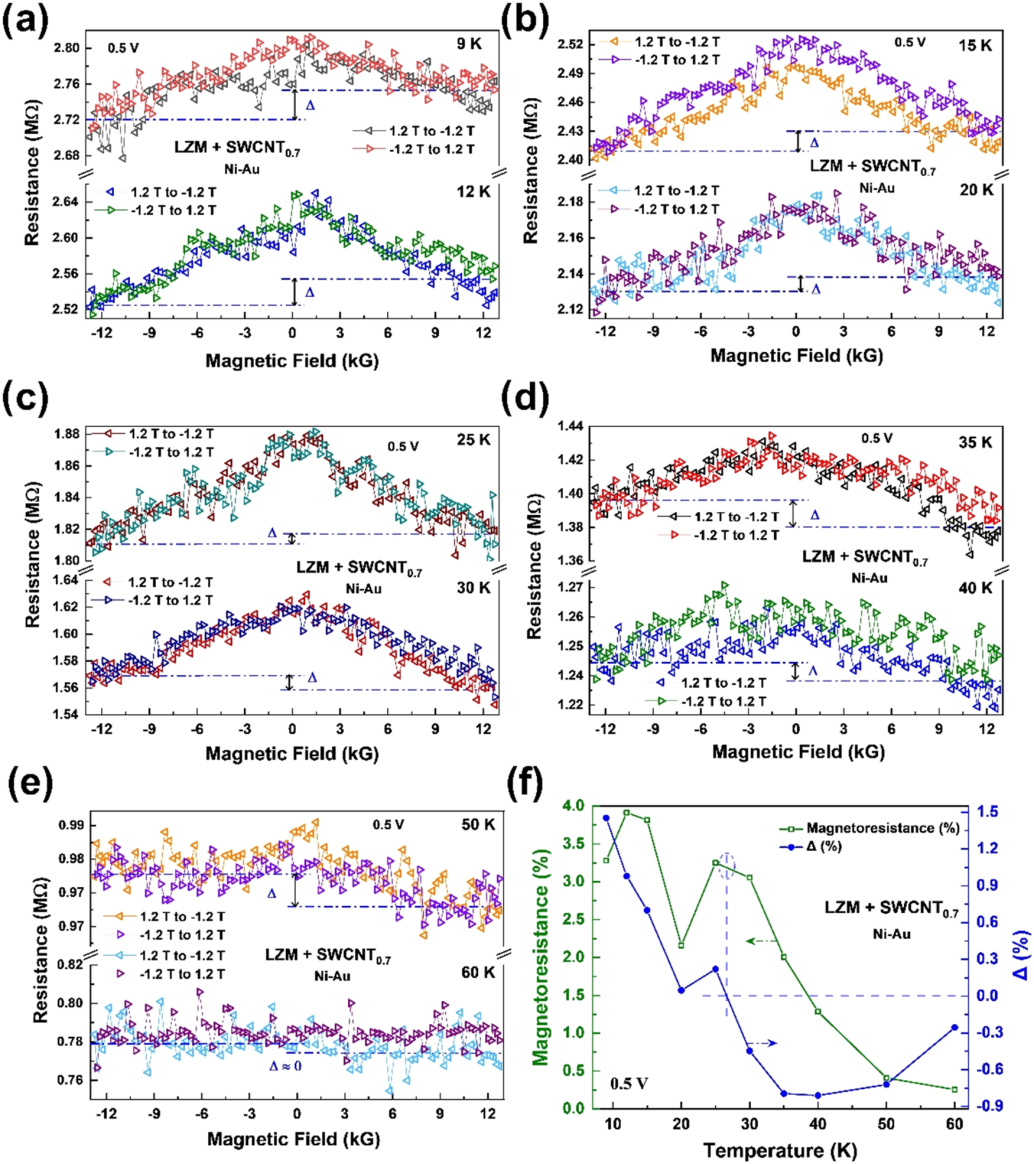
(a–e) Asymmetric MR of SWCNT@CLLC crystals (SWCNT concentration: 0.7 mg/mL, tubes coated with Fmoc-FF-L) with Ni–Au contacts at representative temperatures (applied bias is 0.5 V in all cases). Note that the MR asymmetry Δ flips as the temperature is increased. (**f)** Temperature dependence of background MR and Δ.

Figure 5a–e shows the MR data from SWCNT@CLLC (0.7 mg/mL) samples with Ni–Au contacts. The background MR and its nonmonotonic dependence on temperature (double peak feature) observed before are still present, as summarized in Figure 5f. In addition, the MR responses are asymmetric, resulting in nonzero Δ (∼1.5%). This asymmetry could be attributed to the presence of CISS-induced spin polarization in the nanotubes, which experience different transmission probabilities when Ni magnetization is reversed. As noted, the chiral matrix is insulating, so the asymmetry in the MR cannot be explained by the CISS effect of the matrix itself. Interestingly, the temperature evolution of Δ shows a zero-crossing and, hence, flipping of spin polarization, before it completely disappears at ∼60 K. Temperature evolution of Δ is summarized in Figure 5f. Especially noteworthy is that the zero-crossing of Δ and the second MR peak occur in the same temperature range. Similar features have been observed in SWCNT@CLLC (SWCNT concentration: 0.5 mg/mL) samples as well (Figure S3).

**Figure 6:**
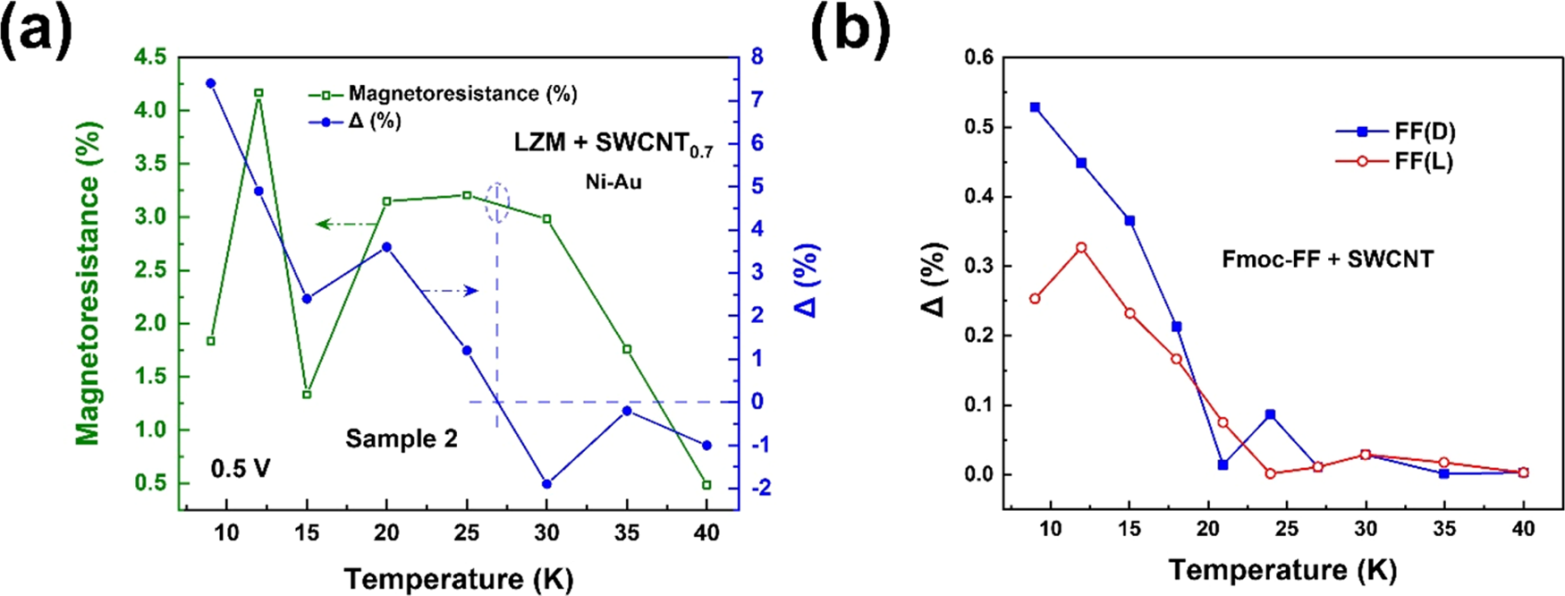
(a) Temperature dependence of background MR and Δ for a second SWCNT@CLLC (SWCNT concentration: 0.7 mg/mL, tubes coated with Fmoc-FF-L) sample. (b) Temperature dependence of the CISS signal Δ for Fmoc-FF + SWCNT samples (i.e., without LZM) in the longitudinal configuration. The decay is monotonic, unlike the SWCNT@CLLC samples reported in Figure 5f and panel (a). As demonstrated in our previous work, the opposite sign of CISS signal Δ for two opposite chiralities appears in the transverse configuration for Fmoc-FF + SWCNT hybrid systems.^27^ At other angles, there is a difference in the magnitude of Δ between the two chiralities.

Data from a second 0.7 mg/mL CNT sample is shown in Figure 6a, which also shows the same features as Figure 5f. It is important to note that this zero-crossing of the CISS signal Δ must be due to LZM because this effect was not observed in samples with Fmoc-FF(L/D) + SWCNT (i.e., no LZM) studied previously.^27,28^ This is illustrated in Figure 6b, showing the temperature dependence of Δ in the longitudinal configuration for Fmoc-FF + SWCNTs, where the decay is monotonic. This difference cannot be attributed to any fundamental difference in the transport mechanism because, as discussed before, the mechanism is the same in both cases. As a consequence of this reversal, the CISS signal Δ remains nonzero up to ∼60 K in SWCNT@CLLC samples, whereas it goes to zero around 25–30 K in the Fmoc-FF(L/D) + SWCNT samples (Figure 6b).^27,28^

As established in Figure 3, charge transport via embedded SWCNT networks is due to phonon-assisted hopping. In such cases, negative background MR, as observed in Figures 4a–c and 5a–e, can have several possible origins: (a) quantum interference between forward hopping paths,^47,48^ (b) quantum interference between forward and backward hopping paths,^49^ (c) enhancement of density of states of Landau levels in the presence of magnetic field,^50^ or (d) magnetic field induced Zeeman splitting.^51^ However, as previous studies on chiral-functionalized SWCNTs have indicated, the mechanism (b) is the most likely candidate.^26,27,52^ In this mechanism, the applied magnetic field breaks the time-reversal symmetry between forward and backward hopping paths and increases the localization length, which results in a negative MR.^49^ We note that this mechanism is reminiscent of the weak-localization effect that manifests in weakly disordered metallic systems.^53^ If the background MR is due to quantum interference effects,^49^ it is expected to disappear gradually as temperature is increased.^49,53^ Generally, such decays are monotonic for bare SWCNTs.^34−36^ However, in this case, we observe two distinct peaks before it disappears (Figures 4d, 5f, and 6a), which was also observed before for Fmoc-FF-SWCNT samples.^27^ The CISS signal (Δ), on the other hand, decays monotonically with temperature for Fmoc-FF + SWCNTs (Figure 6b)^27^ due to spin scattering effects present in SWCNTs.^54^ What is new in the present case of SWCNT@CLLC is the observation of zero-crossing of Δ in the 20–30 K range (Figures 5f and 6a), which indicates spin-flipping within the nanotubes. We also observe that the second MR peak and zero-crossing and spin-flipping occur in the same temperature range. The above features can be explained qualitatively by invoking a key property of CISS, in which spin polarization (***s***) is “locked” with the momentum (***p***) direction.^20,21^ Therefore, in one-dimensional (1D) systems such as nanotubes, any carrier backpropagation (or momentum reversal) event must be accompanied by a simultaneous spin flip. This imposes a constraint on carrier backpropagation because most of the momentum scattering processes, including phonons, conserve spin, and hence, these events are suppressed in CISS systems. In the present case, the CISS-induced spin signal (Δ) is maximum at the lowest temperature (∼10 K). In that range, due to the suppression of carrier backpropagation, interference between forward and backward carrier paths is suppressed, which leads to a smaller value of the background MR. As the temperature is increased in the 10–20 K range, spin signal Δ decays monotonically; as a result, backpropagation events become more likely. This tends to increase the background MR signal; however, increased temperature tries to reduce this effect due to inelastic phonon scattering. Thus, the background MR signal is determined by these two competing processes. This explains the first MR peak around ∼15 K, as well as the second MR peak in the 20–40 K range, where the spin polarization crosses zero. Beyond this range, the monotonic decrease in MR is due to the combined effect of the (reverse) spin polarization as well as increasing temperature. Thus, the multiple MR peaks (Figures 4d, 5f, and 6a) could be attributed to an interplay between quantum interference and CISS-induced spin polarization that suppresses carrier backpropagation. These features have also been observed in SWCNT@CLLC (SWCNT concentration: 0.5 mg/mL) samples (Figure S3f).

**Figure 7:**
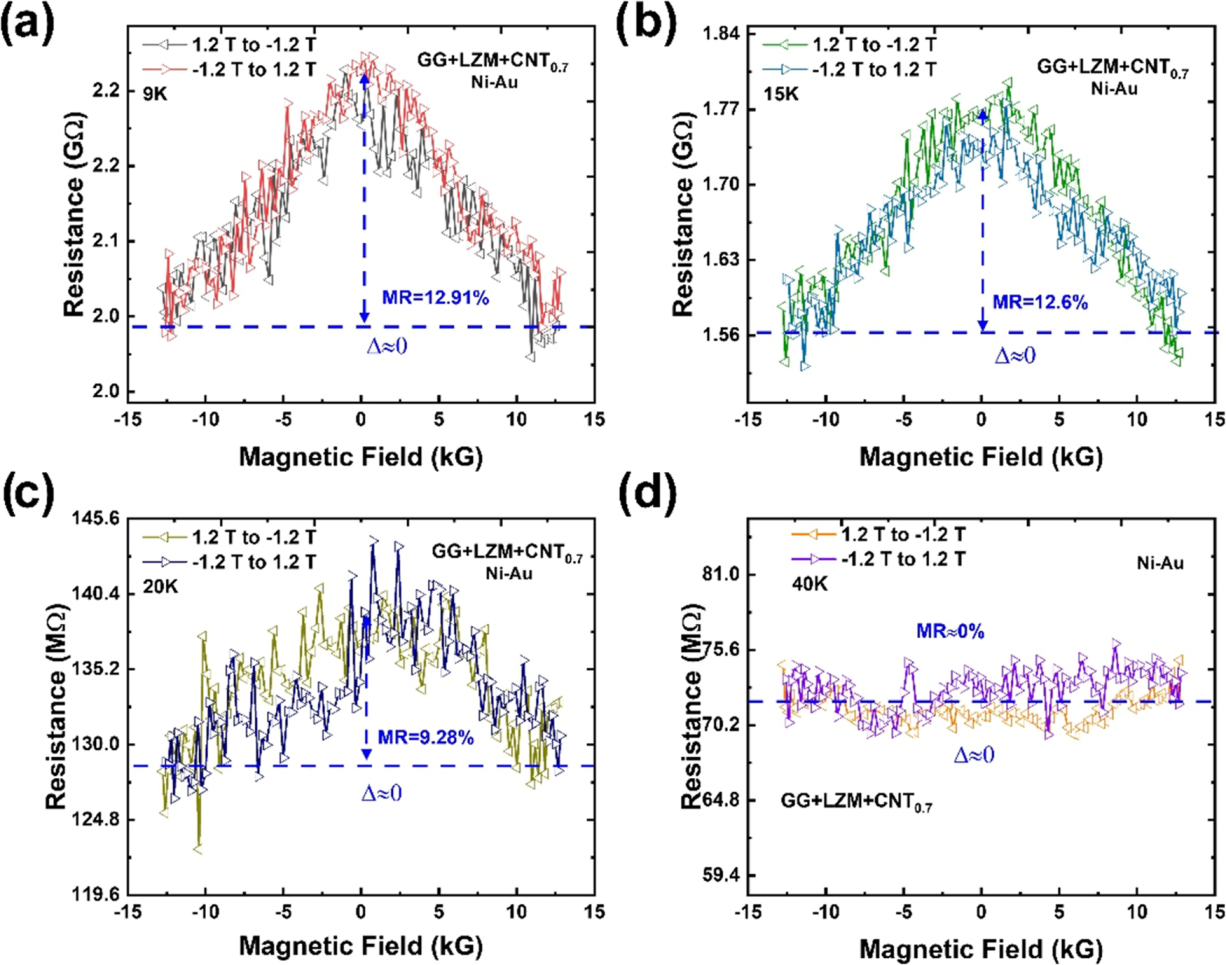
(a–d) Symmetric MR of SWCNT@CLLC crystals (SWCNT concentration: 0.7 mg/mL, tubes coated with achiral Fmoc-GG) with Ni–Au contacts at representative temperatures. The background MR decreases monotonically with temperature.

As mentioned before, the reversal of the CISS signal Δ and its persistence at higher temperatures could be attributed to the presence of the LZM medium. To clarify the role of LZM, we studied the CISS response of SWCNT@CLLCs in which SWCNTs are coated with achiral Fmoc-GG (Figure 7).

Surprisingly, no CISS signal Δ is observed, even though LZM is present. This indicates that the transfer of chirality to the SWCNTs is only exerted by the peptides (directly attached to the tube walls, as shown in Figure 1f) and the role of LZM (the outer layer) is to compress the peptide with the SWCNTs and enhance their interaction. This way, LZM exerts a synergistic influence on the CISS signal, even though it does not interact with the CNTs directly. The background MR, in this case, does not show any double peak feature; instead, it decays monotonically with temperature. This is expected considering the previous explanation, since no CISS effect is present in this case as Fmoc-GG is achiral. The similarity of the transport properties of Fmoc-FF + SWCNTs^27^ with SWCNT@CLLCs (Figure 3) supports the hypothesis that CISS must be induced by Fmoc-FF alone. This control experiment also confirms that the origin of the MR asymmetry discussed earlier must arise from the medium chirality rather than any other artifact of the experiment.

**Figure 8:**
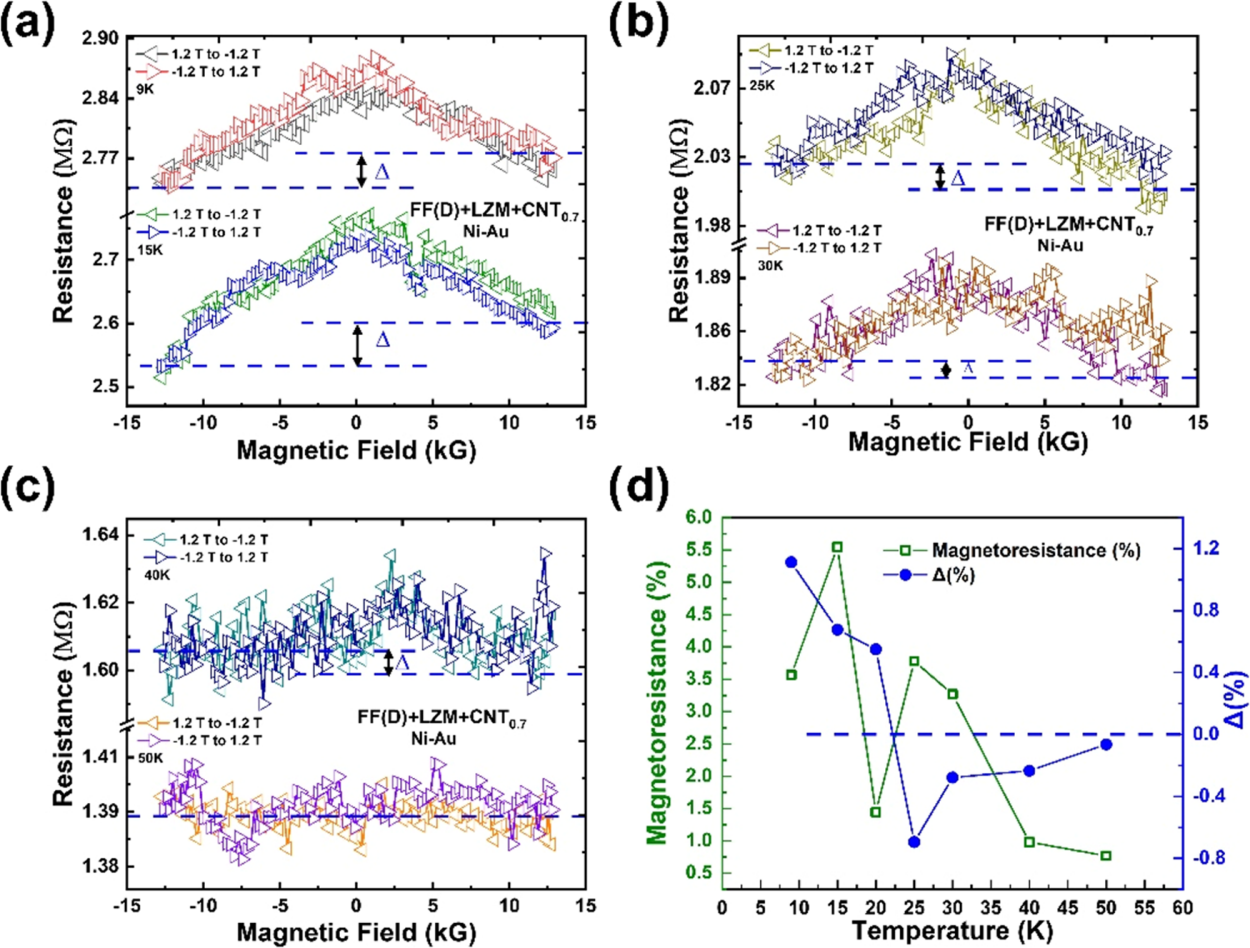
(a–c) Asymmetric MR of SWCNT@CLLC crystals (SWCNT concentration: 0.7 mg/mL, tubes coated with Fmoc-FF-D) with Ni–Au contacts at representative temperatures. Note that the MR asymmetry Δ flips as the temperature is increased. (d) Temperature dependence of background MR and Δ.

The samples discussed in Figures 3–5 used the l-chiral variant of Fmoc-FF as the inner layer. We also replaced this layer with the d variant, and the results are shown in Figure 8. Qualitatively, the l- and d-MR data (Figures 5 and 8) are the same. This is attributed to the longitudinal measurement configuration discussed before, where Fmoc-FF-L and -D show the same sign of CISS. Thus, these results are consistent with the previous studies^27,28^ and support the hypothesis that Fmoc-FF interacts directly with SWCNTs. Comparing the Δ values of SWCNT@CLLC (Figures 5f, 6a, and 8) with that of Fmoc-FF + SWCNT (Figure 6b), we see that the CISS signal is roughly an order of magnitude stronger when LZM is present. This further supports the synergistic role played by the two chiral sources, even though the outer LZM layer does not directly interact with the CNTs.

Temperature-dependent sign reversal of the CISS signal Δ is an interesting observation. As discussed before, there exist two chiral environments: one from LZM (l-chiral) and another from Fmoc-FF (l- or d-chiral). Additionally, stacking of Fmoc-FF gives rise to polymeric fibers that also present chirality.^55,56^ This chirality is known as “supramolecular chirality” and is more sensitive to the environment.^1,2^ Similarly, the stacking of lysozyme units within the crystal structure also develops a supramolecular chirality. Thus, the nanotubes experience not only the intrinsic chirality from the building blocks of protein and peptide (amino acids) but also the supramolecular chirality originating from the stackings of both monomers. We have previously shown that in the case of Fmoc-FF, supramolecular chirality is responsible for the CISS effect.^28,29^ A change in these supramolecular structures with respect to the interaction with CNTs as a function of the temperature could be the likely origin of the observed reversal of Δ. We note that a similar change in molecular structure with temperature and concomitant spin flip has been observed in peptide monolayers.^57^ In this respect, it is known that proteins within the crystal tend to slightly expand when going from lower to higher temperatures, which in this case could result in a change in the interaction with CNTs.^58^ In addition, it has been proposed theoretically that spin-dependent electron–phonon coupling, originating from spin–orbit interaction, can introduce exchange splitting between the spin channels in chiral structures, resulting in CISS.^59^ Considering that CNTs have considerable spin–orbit interaction due to their cylindrical geometry,^60−62^ such phonon-assisted mechanism could also play a role in the present case.

## 3 Conclusions

In conclusion, we have shown that the synergistic influence of multiple supramolecular chiral media can induce nontrivial features in the CISS effect that are not observed in the homochiral case. A detailed comparison between both cases has revealed three nontrivial changes: (a) enhancement of the CISS signal Δ due to a synergistic role played by multiple chiral sources, (b) sign reversal of the CISS signal as a function of temperature, and (c) persistence of the CISS signal at higher temperatures. CISS-induced spin polarization is intricately linked with carrier transport processes in such systems, which manifests as nonmonotonicity in the temperature dependence of quantum interference effects. The broad composition and structural diversity of proteins, together with the economic and easy access to many of them, offer a unique opportunity to develop a multitude of advanced materials with tunable CISS properties, which can add novel spintronics functionalities in the realm of bioelectronics.^63^

## 4 Experimental Section

### 4.1 Hydrogel Control Preparation

*N*-Fluorenylmethoxycarbonyl-l-diphenylalanine (Fmoc-L-FF) was purchased from Bachem Co., Switzerland, and used without further purification. *N*-Fluorenylmethoxycarbonyl-d-diphenylalanine (Fmoc-D-FF) was purchased from LifeTein, and used without further purification. *N*-Fluorenylmethoxycarbonyl-diglycine (Fmoc-GG) was purchased from Fluorochem, U.K., and used without further purification. Fmoc-FF (l- or d-chiral) and Fmoc-GG peptides were weighed separately into a vial and deionized water was added to obtain a final concentration of 10 mM. This suspension was then sonicated (in an HSt Powersonic 405-ultrasonic bath) for 1 h. A 0.5 MNaOH solution was then added dropwise, and an aqueous basic solution was obtained (pH ≈ 10.5). Gelation was induced by adding 2 mol equiv of glucono-δ-lactone (GdL), and the solution was mixed by vortexing. Full gelification was achieved after 12 h at room temperature.

### 4.2 Composite Hydrogel Preparation

A suspension of SWCNTs was prepared by weighing 0.7 mg of SWCNTs (∼95% semiconducting with ∼41% having (6,5) chirality; ≥95% carbon basis, i.e., ≥95% as carbon nanotubes; 0.78 nm average diameter; CAS: 308068-56-6, provided by Sigma- Aldrich: https://www.sigmaaldrich.com/ES/es/product/aldrich/773735) into a vial tube. The carbon nanotubes were suspended in 1 mL of an aqueous basic solution of Fmoc-FF (l- or d-chiral) or Fmoc-GG 0.5% w/v (prepared as above). The suspension was then sonicated for 2 h in a cold ultrasonic bath and centrifuged for 5 min at 10.000 rpm (Sigma 1–14 centrifuge). Finally, the supernatant was collected carefully. Gelation was then carried out following the same method described above for the hydrogel control. Protocol to produce Fmoc-FF hydrogels having 0.5 mg of SWCNTs or less can be found in our previous work.^32^

### 4.3 Crystallization Protocol

A commercial lysozyme (HEWL chicken) was used as a lyophilized powder from Sigma and dissolved in 50 mM sodium acetate of pH 4.5. The concentration of the lysozyme was determined spectrophotometrically at 280 nm using ε = 2.65 mL/(mg·cm). Crystallization experiments were carried out using the counterdiffusion technique in the two-chamber method in Eppendorf tubes following the protocol described elsewhere.^9^ Composite hydrogels were dialyzed against 100 μL of Milli-Q water for 1 week, changing the water every day. Next, 100 μL of the buffered protein solution, at 120 mg/mL, was added on top of the hydrogels, and they were allowed to diffuse at 20 °C for 1 week. After that time, the solution was replaced with 100 μL of the precipitant solution (5–7% w/v sodium chloride in 50 mM sodium acetate, pH 4.5) and allowed to diffuse at 20 °C. Eppendorf tubes were kept in incubators at 20 °C and periodically observed with the aid of an optical microscope. The first crystals appeared earlier than 24 h and reached their maximum size in a week. The largest dimension of the grown crystals was 60 μm.

Crystals were observed by optical microscopy after extraction in an isotonic solution. Optical images were recorded using the Image-Focus-α software of the Nikon AZ100 microscope with a zoom of 4 × 3 x 0.6.

Lysozyme has been extensively studied also in solution, and there are many CD spectra already published, i.e., at several pH values and in the presence of methanol,^64^ in the presence of surfactants,^65^ in comparison to its amyloid state,^66^ etc.

### 4.4 Cross-Linking Protocol

CLLC was obtained by diffusing the cross-linker throughout the crystallization chamber.^32^ The precipitant solution was replaced by an isotonic solution containing 5% v/v glutaraldehyde as a cross-linking agent and allowed to diffuse at 20 °C for 24 h. After that time, the hydrogel containing the crystals was extracted into a Petri dish filled with Milli-Q water. The crystals were removed from the gel with the aid of a brush and microtools, collected, and stored in Milli-Q water in an Eppendorf tube at 20 °C.

### 4.5 Device Fabrication for Transport Experiments

The SWCNT@CLLC crystals were embedded in epoxy resin EMbed-812 (Electron Microscopy Sciences) and placed in a 60 °C oven for 48–72 h for polymerization. Serial ultrathin sections (∼100–900 nm range) were sliced sequentially by a Reichert Ultracut S microtome (Leica Microsystems, Germany). Next, the sections were transferred on Au–Au and Ni–Au electrodes (electrode thickness ∼100 nm, electrode gap ∼2 μm) photolithographically patterned on the SiO_2_ (500 nm)/Si wafer. Finally, the devices were vacuum annealed (200 °C for 30 min) to improve electrical contacts between the electrodes and the transferred sections.

### 4.6 Raman Spectroscopy

For Raman characterization, the sections were placed on glass substrates. Data was collected using Thermo Scientific DXR2 Raman Microscope, 100X confocal, 532 nm laser, and 5 mW power.

### 4.7 Transmission Electron Microscopy

The crystals were studied with a Carl Zeiss POUND 120 PLUS. The cross-linked lysozyme crystals (CLLCs) with nanotubes (SWCNT@CLLC) were dehydrated with ethanol and embedded in Embed-812 resin. Ultrathin sections (50–70 nm) were prepared using a Reichert Ultracut S microtome (Leica Microsystems GmbH, Wetzlar, Germany), after which the sections were deposited on copper grids.

### 4.8 Scanning Electron Microscopy

SEM Images of SWCNT@CLLCs cut with a scalpel were put on an adhesive surface, coated with a fine carbon layer, and examined by SEM using a field emission scanning electron microscope (FESEM) GEMINI, LEO 1500 Carl Zeiss.

### 4.9 Circular Dichroism

Peptide basic solutions and hydrogels were recorded using a Jasco J-815 spectrophotometer with a 150 W xenon lamp of 150 W. The mixtures were jellified into a 0.1 mm quartz cell (Hellma 0.1 mm quartz SuprasilR). The spectra were obtained from 220 to 350 nm with a 1 nm step and 1 s integration time per step at 25 °C.

## Supporting Information Available

Current–voltage (*I*–*V*) characteristics of SWCNT@CLLC crystals (SWCNT concentration: 0.5 mg/mL) (Figure S1); fitting of the *I*–*V* data of SWCNT@CLLC crystals with the VRH model (Figure S2); asymmetric MR of SWCNT@CLLC (SWCNT concentration: 0.5 mg/mL) and temperature dependence (Figure S3) (PDF)

## Footnotes

a MR values are calculated as [*R*(*B* = 0 kG) – *R*(*B* = −12 kG)]/*R*(*B* = −12 kG) × 100%.
