# Supplementary material for: Efficient Transfer of Chirality in Complex Hybrid Materials and Impact on Chirality-induced Spin Selectivity
Source: Chem Mater. 2024 Nov 20;36(23):11449–61. doi: 10.1021/acs.chemmater.4c02108 (PMC11635975; doi:10.1021/acs.chemmater.4c02108)
Supplement: Supplementary file 1 — cm4c02108_si_001.pdf [file cm4c02108_si_001.pdf]

Supplementary Information

Efficient Transfer of Chirality in Complex Hybrid Materials and Impact on Chirality-induced Spin Selectivity

Md Anik Hossain,[a,#] Sara Illescas-Lopez,[b] Md Wazedur Rahman,[a,c,#] Mari C. Mañas Torres,[b] Rafael Contreras-Montoya,[d] Seyedamin Firouzeh,[a] José A. Gavira,[e]* Luis Álvarez de Cienfuegos[b,f]* and Sandipan Pramanik[a]*

[a] Department of Electrical and Computer Engineering, University of Alberta, Edmonton, AB T6G 1H9 Canada.

[b] Universidad de Granada (UGR), Departamento de Química Orgánica, Unidad de Excelencia Química Aplicada a Biomedicina y Medioambiente (UEQ), C. U. Fuentenueva, Avda. Severo Ochoa s/n, E-18071 Granada, Spain.

[c] National Research Council Canada, Edmonton, AB T6N 1E4, Canada.

[d] Nanoscopy-UGR Laboratory, Facultad de Farmacia, Campus de Cartuja, 18071 Granada, Spain.

[e] Laboratorio de Estudios Cristalográficos, Instituto Andaluz de Ciencias de la Tierra (Consejo Superior de Investigaciones Científicas), Avenida de las Palmeras 4, 18100 Armilla, Granada, Spain.

[f] Instituto de Investigación Biosanitaria ibs.GRANADA Av. de Madrid, 15, 18016, Granada, Spain.

[#] Equal contributions.

* Corresponding authors (j.gavira@csic.es; lac@ugr.es; spramani@ualberta.ca)

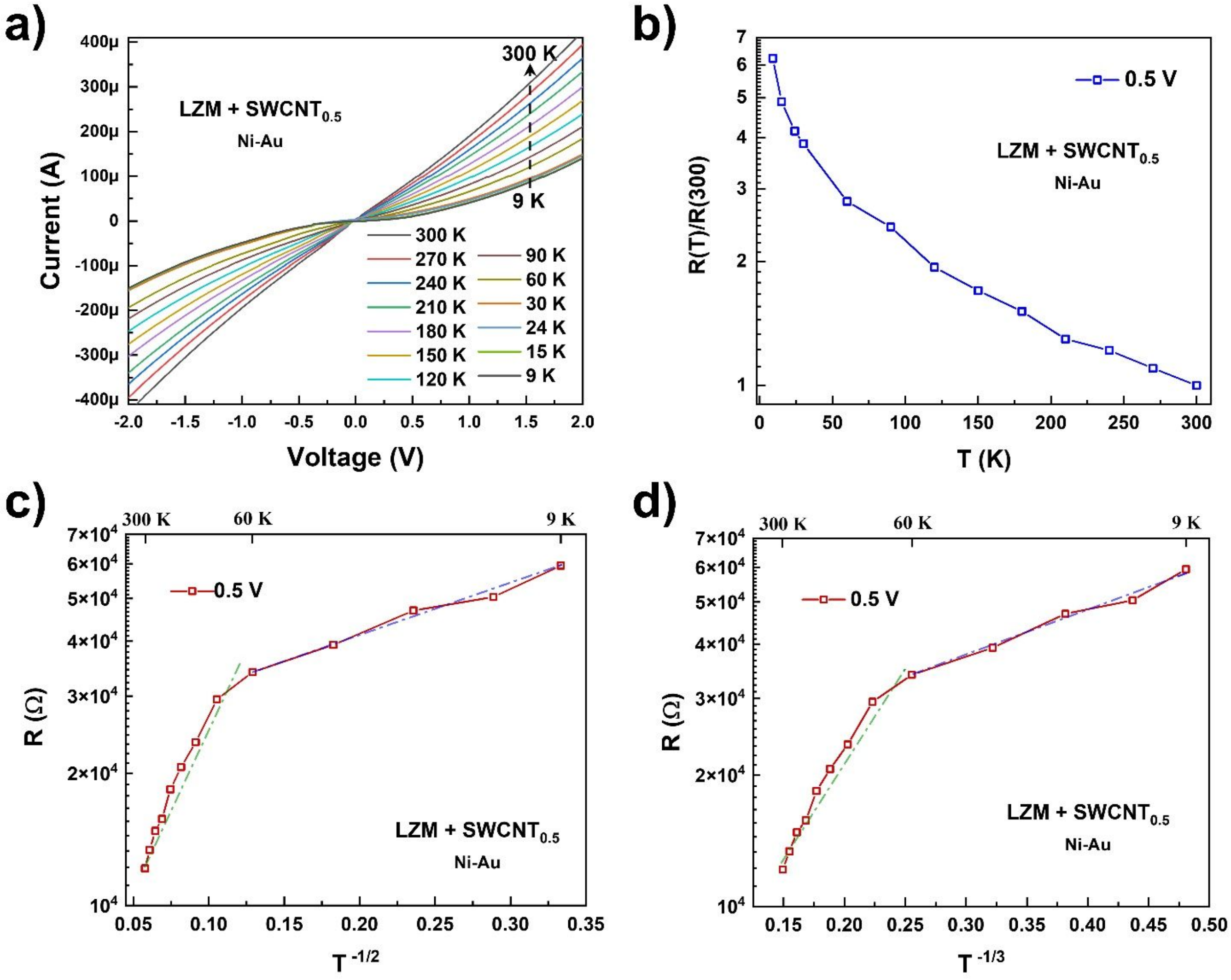

Figure S1. (a) Current-voltage (*I-V*) characteristics of SWCNT@CLLC crystals (SWCNT concentration 0.5 mg/ml, tubes coated with Fmoc-FF-L) at $B = 0$ using Ni-Au electrodes. (**b)** Temperature-dependence of the normalized resistance. (**c), (d)** Fitting with the VRH model for $d = 1$ and 2 respectively.

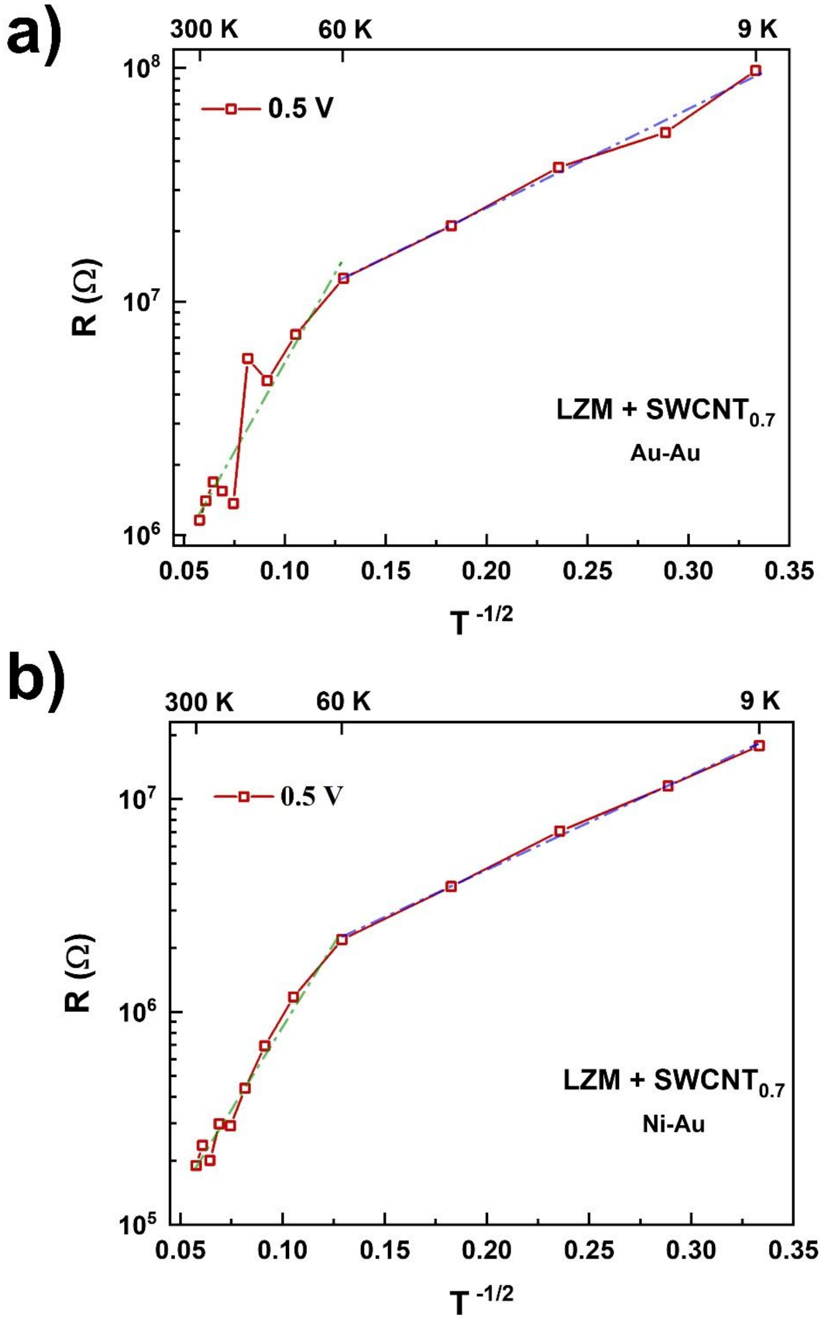

Figure S2. Fitting with the VRH model of SWCNT@CLLC crystals (SWCNT concentration 0.7 mg/ml, tubes coated with Fmoc-FF-L) *I-V* data from Figure 3 (main paper) for $d = 1$. (a) Au-Au and (b) Ni-Au electrodes.

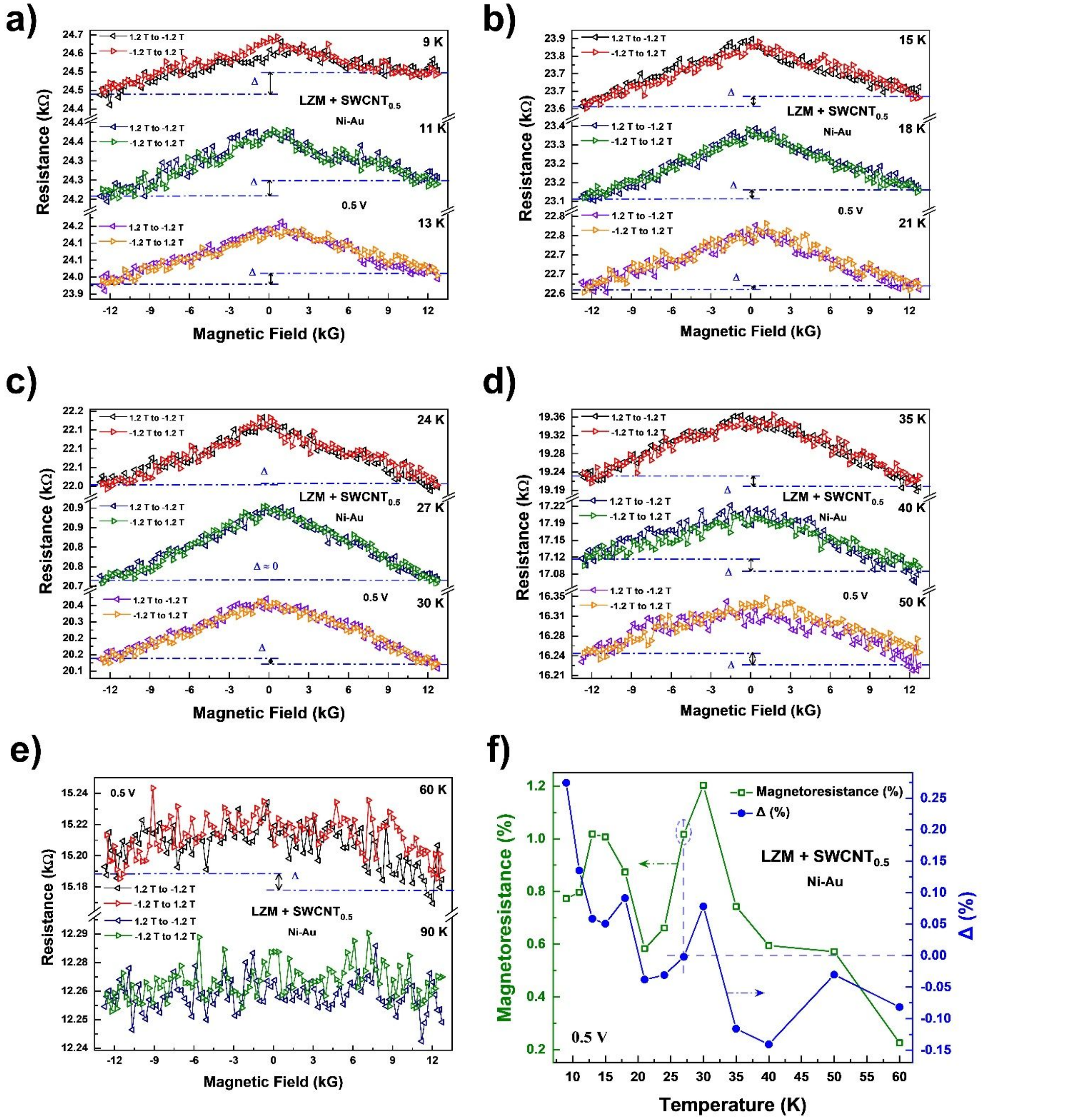

Figure S3. (a)-(e) *Asymmetric* MR of SWCNT@CLLC (SWCNT concentration: 0.5 mg/ml, tubes coated with Fmoc-FF-L) with Ni-Au contacts at different temperatures. Applied bias is 0.5V in all cases. Note that the MR asymmetry Δ flips as temperature is increased. (**f**) Temperature-dependence of background MR and Δ.
